# A scoring system based on novel biomarkers and clinical risk factors to predict invasive candidiasis in immunocompetent critically ill patients

**DOI:** 10.3389/fmicb.2023.1097574

**Published:** 2023-03-09

**Authors:** Wen Li, Gang Chen, Fengyu Lin, Hang Yang, Yanhui Cui, Rongli Lu, Chao Song, Haitao Li, Yi Li, Pinhua Pan

**Affiliations:** ^1^Department of Respiratory Medicine, National Key Clinical Specialty, Branch of National Clinical Research Center for Respiratory Disease, Xiangya Hospital, Central South University, Changsha, China; ^2^Center of Respiratory Medicine, Xiangya Hospital, Central South University, Changsha, China; ^3^Hunan Engineering Research Center for Intelligent Diagnosis and Treatment of Respiratory Disease, Changsha, China; ^4^National Clinical Research Center for Geriatric Disorders, Xiangya Hospital, Changsha, China; ^5^Clinical Research Center for Respiratory Diseases in Hunan Province, Changsha, China; ^6^Nosocomial Infection Control Center, Xiangya Hospital, Central South University, Changsha, China; ^7^First Department of Thoracic Medicine, Hunan Cancer Hospital, The Affiliated Cancer Hospital of Xiangya School of Medicine, Central South University, Changsha, China

**Keywords:** invasive candidiasis, scoring system, immunocompetent, novel serological biomarkers, critically ill patients

## Abstract

**Background:**

Delayed diagnosis further increases the mortality of invasive candidiasis (IC) in intensive care unit (ICU) patients. This study aimed to develop and validate a score based on novel serological biomarkers and clinical risk factors for predicting IC in immunocompetent ICU patients.

**Methods:**

We retrospectively collected clinical data and novel serological markers on admission to ICU. Multivariate logistic regression was used to identify the risk factors associated with IC, which were adopted to establish a scoring system.

**Results:**

Patients with IC had a higher C-reactive protein-to-albumin ratio (CAR) and neutrophil-to-lymphocyte ratio (NLR) and lower prognostic nutritional index than those without IC. The NLR, CAR, sepsis, total parenteral nutrition, 1,3-β-D-glucan (BDG)-positivity, and Sequential Organ Failure Assessment score were identified as independent risk factors for IC by multivariate logistic regression analysis and entered into the final scoring system. The area under receiver operating characteristic curve of the score were 0.883 and 0.892, respectively, in the development and validation cohort, higher than Candida score (0.883 vs.0.730, *p* < 0.001).

**Conclusion:**

We established a parsimonious score based on NLR, CAR, BDG-positivity, and clinical risk factors, which can accurately identify IC in ICU patients to give treatment on time and reduce mortality.

## Introduction

Invasive candidiasis (IC) refers to a bloodstream (termed candidemia) and deep-seated infection with *Candida* spp. (Pappas et al., 2016; Bassetti et al., 2019). Candidemia is the second most common nosocomial bloodstream infection, with a mortality rate of 49% (McCarty et al., 2021). Approximately 1/3 of candidemia cases occur in the intensive care unit (ICU), and IC is the most common fungal disease in ICU patients (Logan et al., 2020). Patients admitted to the ICU have numerous risk factors significantly associated with IC, such as wide use of broad-spectrum antibiotics (Thomas-Ruddel et al., 2022), prolonged duration of corticosteroid use, post-aggressive immune paralysis (Jung et al., 2021), and higher utilization of intravenous catheters (Kullberg and Arendrup, 2015; Pappas et al., 2018). The overall mortality rate of ICU-associated IC is up to 55%, and has even been documented at 98% when septic shock develops (Kullberg and Arendrup, 2015). Previous studies and guidelines have mostly focused on immunodeficient patients, such as those with hematological malignancies and solid organ transplants (Donnelly et al., 2020). However, most patients in the ICU are immunocompetent at the onset of infection (Bassetti et al., 2021). Therefore, there is a lack of strong evidence to guide early recognition and timely initial antifungal therapy for IC among immunocompetent ICU patients.

Notably, delayed antifungal treatment can further increase the mortality of ICU-related IC, especially when not initiated 24–48 h after the first positive culture (Playford et al., 2016). Most ICU patients have hemodynamic instability, thrombocytopenia, or coagulation disorders, which may complicate tissue sampling. Furthermore, current pathogen detection methods for IC have limitations and cannot achieve timely and accurate diagnosis at the onset of infection. Culture-based techniques require more than 1–2 days to obtain results and have low sensitivity (Clancy and Nguyen, 2013). The most common non-culture-based method, testing for 1,3-β-D-glucan (BDG), has narrow specificity and sensitivity (Lamoth et al., 2012). Multiplex *Candida* real-time polymerase chain reaction (PCR) and metagenomics second-generation gene sequencing have great advantages in etiological diagnosis; however, they have some limitations, such as cost and the need for skilled personnel, and it is difficult to carry out routine etiological examinations (Bassetti et al., 2019). Therefore, the early recognition of IC in ICU patients is problematic. Hence, it is necessary and urgent to construct a tailored, prompt, and easy-to-use diagnostic clinical model.

*Candida* invades the human body and activates innate immune cells to produce many proinflammatory cytokines and chemokines (Pappas et al., 2018). Previous studies have found that plasma levels of inflammatory cytokines [Interleukin-1β (IL-1β), Tumor necrosis factor-α (TNF-α), and Interferon-γ (IFN-γ)] and clinical inflammatory markers [C-reactive protein (CRP) and neutrophils] are significantly increased in patients with IC (Chumpitazi et al., 2014; Akin et al., 2015; Wunsch et al., 2021). Furthermore, several previous studies have shown that patients with IC in the ICU are at a high risk of malnutrition, which is associated with poor clinical prognosis (Piazza et al., 2004; Lee et al., 2021). Therefore, patients with IC in the ICU exhibit extremely severe systemic inflammation and poor nutritional status. Novel serological markers derived from a combination of two or more laboratory indicators, such as the neutrophil-to-lymphocyte ratio (NLR), platelet-to-lymphocyte ratio (PLR), monocyte-to-lymphocyte ratio (MLR), CRP-to-albumin ratio (CAR), and systemic immune-inflammation index (SII), represent inflammation and immune response (Xue et al., 2020). The prognostic nutritional index (PNI) symbolizes the nutritional status of the patients (Xue et al., 2020). However, changes in these novel serological biomarkers and their clinical value in IC remain unclear. In this retrospective study, we aimed to evaluate the associations between novel serological biomarkers, disease severity, and prognosis and to develop a simple predictive scoring system based on clinical risk factors and new biomarkers for the early diagnosis of IC in ICU patients to guide timely initial antifungal therapy.

## Methods

### Study setting and patients enrollment

**Figure 1:**
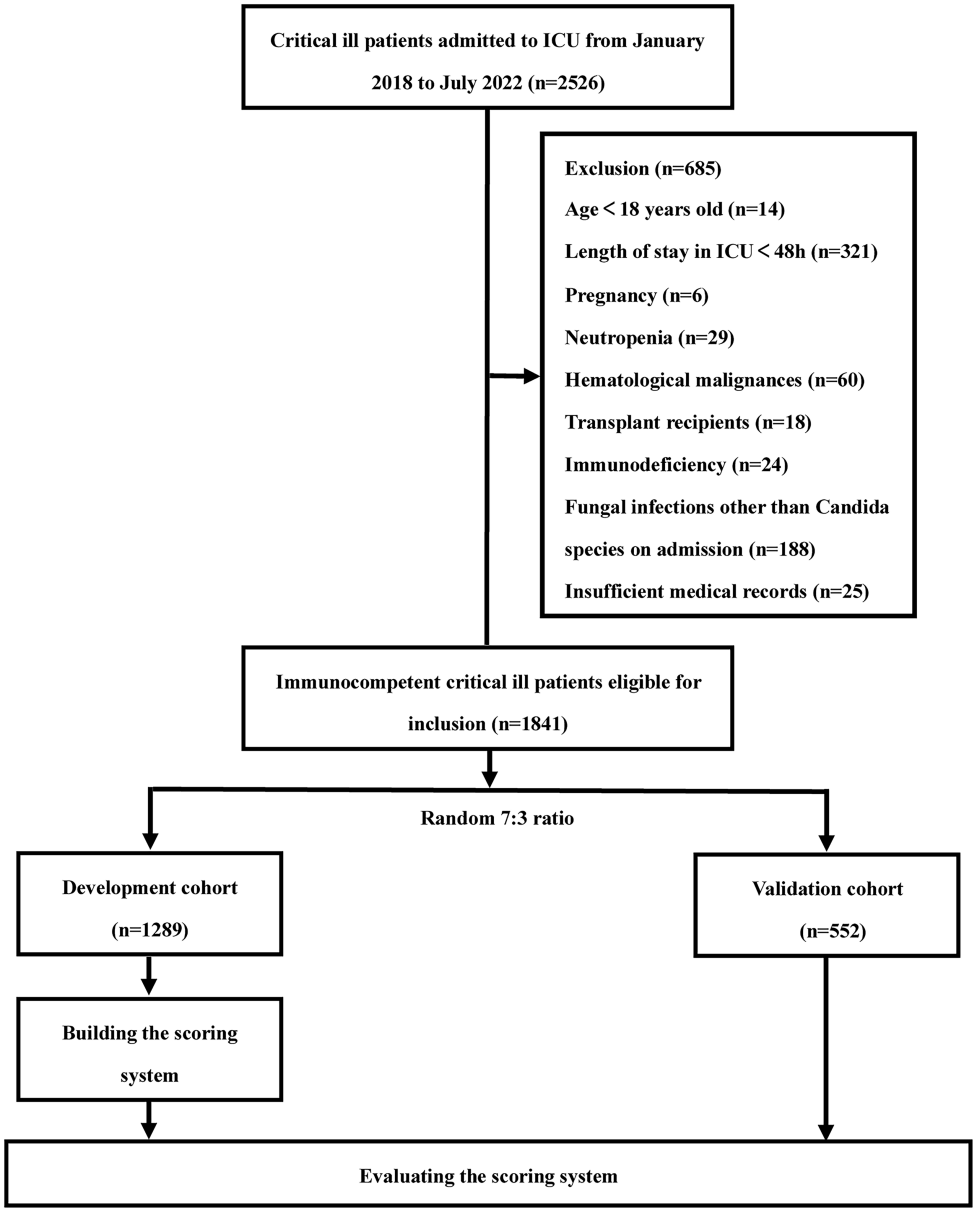
Flow diagram of the study population.

We retrospectively collected data on consecutive patients admitted to the ICU at Xiangya Hospital of Central South University from January 2018 to July 2022. The inclusion criteria were as follows: (1) age ≥ 18 years and (2) critical illness requiring intensive care. We excluded patients with an expected length of stay in the ICU of less than 48 h, pregnancy, fungal infections other than *Candida* species on admission, insufficient medical records and immunocompromised patients [a. transplant recipients (solid organ or hematopoietic stem cell transplant); b. hematological malignancies; c. neutropenia (peripheral absolute neutrophil count <500 cells/mm^3^); d. immunodeficiency (primary immunodeficiency or acquired immunodeficiency caused by immunosuppressive drugs, chemotherapy, and human immunodeficiency virus)]. A total of 1,841 ICU patients participated in the study, including 84 patients with IC. The flowchart of this retrospective study is shown in Figure 1. This study was approved by the Ethics Committee of the Xiangya Hospital, Central South University (No. 202104005). Due to the retrospective nature of the study and the anonymous processing of data prior to analysis, the Ethics Committee waived the requirement for informed consent. The study strictly complied with the Declaration of Helsinki.

### Criteria for diagnosis

IC was diagnosed by experienced respiratory physicians according to the criteria outlined by the European Organization for Research on Treatment of Cancer and Mycology Study Group Education and Research Consortium (EORTC/MSGERC) (Bassetti et al., 2021) based on clinical manifestations consistent with IC, plus one of the following: (1) sterile tissue obtained by needle aspiration or biopsy showing budding cells consistent with *Candida* species by histopathology, cytopathology, or direct microscopic examination; (2) sterile specimens obtained through sterile procedures (such as pleural effusion, ascites, and cerebrospinal fluid) showing *Candida* spp.; or (3) positive peripheral blood culture for *Candida*. A high corticosteroid dose was defined as prednisone ≥20 mg/day (Kluge et al., 2021). A history of broad-spectrum antibiotics means that the patient has received antibiotics for more than 10 days in the last month against both gram-positive and gram-negative bacteria (Gu et al., 2021).

### Clinical data collection

The baseline clinical characteristics and laboratory test results of each patient were extracted from the electronic medical system. A team of experienced respiratory clinicians independently collected, reviewed, and analyzed clinical data. Demographic and clinical characteristics at admission to the ICU included age, sex, smoking status, vital signs (body temperature, heart rate, respiratory rate, and blood pressure), oxygenation index, Sequential Organ Failure Assessment (SOFA score), comorbidities, laboratory test findings (routine blood test, liver and kidney function tests, BDG, and CRP). All baseline laboratory examinations were completed within 24 h after ICU admission. And other IC-associated risk factors include mechanical ventilation methods, vasopressors, continuous renal replacement therapies (CRRT), total parenteral nutrition (TPN), presence of urinary catheter or central venous catheter (CVC), and administration of a high dose of corticosteroid or a broad spectrum of antibiotic. The primary clinical outcome of this study was the 30-day mortality after ICU admission. Secondary outcomes included length of hospital stay, length of ICU stay, and hospitalization expenses.

### New serological markers

These new serological markers are composed of two or more clinical laboratory indicators. The novel markers and their formulas are as follows (Li et al., 2021): (1) CAR = CRP(mg/L) /albumin(g/L), (2) NLR = neutrophil counts(10^9^/L) /lymphocyte counts(10^9^/L), (3) PNI = 10 × albumin(g/L) + 5 × lymphocyte count(10^9^/L), (4) MLR = monocyte counts(10^9^/L) /lymphocyte counts(10^9^/L), (5) PLR = platelet counts(10^9^/L) /lymphocyte counts(10^9^/L), (6) SII = platelet count(10^9^/L) × neutrophil count (10^9^/L) /lymphocyte count (10^9^/L).

### Statistical analysis

Continuous variables were represented as mean ± standard deviation (SD) or median (range) and compared using Student’s *t*-test and Mann–Whitney *U* test. Categorical data were expressed as frequencies and percentages and compared using the chi-square test or Fisher’s exact test. The study population was randomly divided into development and validation cohorts at a ratio of 7:3. Variables demonstrating possible risk factors for IC were transformed from continuous variables into categorical variables using cut-off values generated from receiver operating characteristic (ROC) analysis. Significant variables in the univariate logistic regression analysis were entered into multivariate forward stepwise logistic regression analysis. Final significant indicators were included in the predictive scoring system. The weighed point of each variable was defined by the corresponding β coefficient, which was divided by the absolute value of the smallest β coefficient and rounded to the nearest integer. The area under the curve (AUC) was used to evaluate the performance of the scoring system. The calibration was checked by plotting the actual probabilities and model-predicted probabilities of the IC. The fitness of the model was calculated using the Hosmer–Lemeshow test (*p* > 0.05, good fitness). The aforementioned validation methodologies were conducted in both the development and validation cohorts. The optimal cutoff values were determined by calculating the Youden index. The sensitivity, specificity, positive predictive value (PPV), negative predictive value (NPV), positive likelihood ratio (LR^+^), and negative likelihood ratio (LR^−^) were calculated. All statistical tests were two-tailed, and differences were considered statistically significant at *p* < 0.05. Statistical analysis was performed using SPSS (version 26.0; SPSS Company, Chicago, IL, United States) and R Statistical Software 4.1.3 (R Foundation for Statistical Computing, Vienna, Austria). Figures were drawn using GraphPad Prism version 9.00 software and R Statistical Software 4.1.3.

## Results

### Demographic and clinical characteristics of the study population

A total of 1,841 ICU patients were enrolled in the study, including 1,289 in the development cohort and 552 in the validation cohort (Figure 1). The demographic and clinical characteristics of the two cohorts are shown in Supplementatry Table S1. There were no significant differences in the baseline clinical characteristics and novel serological marker levels between the two cohorts. The overall incidence of IC was 4.56% (84/1,841).

Patients in the IC group had higher rates of diabetes mellitus (DM), sepsis, and solid tumor than those in the non-IC group (Table 1; *p* < 0.05). The mean arterial pressure and oxygenation index were significantly lower in the IC group. Higher proportions of patients with IC received life-sustaining treatments, indwelling catheters, immunoglobulins, and broad-spectrum antibiotics than patients without IC (*p* < 0.05). With regard to disease severity, patients with IC had significantly higher SOFA scores than those without IC (*p* < 0.001). With reference to clinical outcomes, the 30-day mortality rate was significantly higher in the IC group than in the non-IC group (21.43% vs. 13.15%, *p* = 0.03). Furthermore, patients with IC had a longer hospital stay (14.5 vs. 9 days, *p* < 0.001) and ICU stay (10 vs. 7 days, *p* < 0.001) than those without IC. In addition, the hospitalization cost of patients with IC was significantly higher than that of patients without IC.

**Table 1:** Baseline characteristics in immunocompetent critically ill patients.

Variables	Total (*n* = 1841)	IC group (*n* = 84)	Non-IC group (*n* = 1757)	*P* value
Age, median (IQR), years	67.00	68.00	67.00	0.356
	(56.00–74.00)	(57.00–75.00)	(56.00–74.00)	
Male, *n* (%)	1,342 (72.90%)	67 (79.76%)	1,275 (72.57%)	0.147
Smoke history, *n* (%)	1,100 (59.75%)	62 (73.81%)	1,038 (59.08%)	0.007
Comorbidities, *n* (%)				
COPD	564 (30.64%)	13 (15.48%)	551 (31.36%)	0.002
Diabetes mellitus	427 (23.19%)	27 (32.14%)	400 (22.77%)	0.047
Sepsis	396 (21.51%)	48 (57.14%)	348 (19.81%)	<0.001
Pulmonary tuberculosis	278 (15.10%)	12 (14.29%)	266 (15.14%)	0.831
Bronchiectasis	233 (12.66%)	8 (9.52%)	225 (12.81%)	0.377
Chronic renal failure	224 (12.17%)	9 (10.71%)	215 (12.24%)	0.677
Interstitial lung disease	181 (9.83%)	11 (13.10%)	170 (9.68%)	0.304
Rheumatic immune disease	176 (9.56%)	12 (14.29%)	164 (9.33%)	0.132
Lung cancer	172 (9.34%)	6 (7.14%)	166 (9.45%)	0.478
Other solid tumor	131 (7.12%)	16 (19.05%)	115 (6.55%)	<0.001
Asthma	81 (4.40%)	1 (1.19%)	80 (4.55%)	0.142
Hepatic failure or cirrhosis	39 (2.12%)	1 (1.19%)	38 (2.16%)	0.546
Vital signs on admission to ICU				
Body temperature (°C)	36.80	36.80	36.80	0.223
	(36.50–37.00)	(36.60–37.25)	(36.50–37.00)	
Respiratory rate (times per minute)	23.00	24.00	23.00	0.313
	(21.00–26.00)	(20.25–26.00)	(21.00–26.00)	
Heart rate (times per minute)	92.00	98.00	92.00	0.028
	(80.00–105.00)	(84.25–109.25)	(80.00–104.50)	
Mean arterial pressure (mmHg)	91.00	86.17	91.33	0.008
	(81.33–101.17)	(74.75–98.00)	(81.67–101.33)	
Oxygenation index (mmHg)	215.15	167.97	217.24	<0.001
	(144.26–311.11)	(112.42–249.90)	(146.67–314.47)	
Life-sustaining treatments, *n* (%)				
Non-invasive mechanical ventilation	358 (19.45%)	8 (9.52%)	350 (19.92%)	0.019
Invasive mechanical ventilation	804 (43.67%)	63 (75.00%)	741 (42.17%)	<0.001
Vasopressor	589 (31.99%)	52 (61.90%)	537 (60.56%)	<0.001
CRRT	170 (9.23%)	25 (29.76%)	145 (8.25%)	<0.001
TPN	215 (11.68%)	23 (27.38%)	192 (10.93%)	<0.001
Surgery	84 (4.56%)	3 (3.57%)	81 (4.61%)	0.236
Indwelling catheter, *n* (%)				
Urinary catheter	1,129 (61.33%)	71 (84.52%)	1,058 (60.22%)	<0.001
CVC	583 (31.67%)	53 (63.10%)	530 (30.17%)	<0.001
Drug therapy, *n* (%)				
High dose of corticosteroid	776 (42.15%)	36 (42.86%)	740 (42.12%)	0.893
IVIG	142 (7.71%)	21 (25.00%)	121 (6.89%)	<0.001
Broad-spectrum antibiotics>10 days	1,201 (65.24%)	76 (90.48%)	1,125 (64.03%)	<0.001
Disease severity score				
SOFA score, median (IQR)	4.00	5.00	4.00	<0.001
	(2.00–5.00)	(3.25–7.00)	(2.00–5.00)	
Fungal infection marker				
BDG positivity, *n* (%)	139 (7.55%)	41 (48.81%)	98 (5.58%)	<0.001
Clinical outcomes				
30-day mortality, n (%)	249 (13.53%)	18 (21.43%)	231 (13.15%)	0.030
Length of hospital stay of survivors (days)	9.00	14.50	9.00	<0.001
	(6.00–16.00)	(8.00–22.00)	(6.00–16.00)	
Length of ICU stay of survivors (days)	7.00	10.00	7.00	<0.001
	(4.00–10.00)	(6.00–16.00)	(4.00–10.00)	
Hospitalization expenses	56460.06 (34266.20–107862.04)	133987.72 (76673.19–198379.30)	55192.18 (33723.12–102160.85)	<0.001

Data were presented as median (IQR) and *n* (%). *p* values were calculated by Mann–Whitney *U* test, or Chi-square test, as appropriate. *P* values indicated differences between the IC and non-IC patients. COPD, chronic obstructive pulmonary disease; IVIG, intravenous immunoglobulin; IQR, interquartile range.

### Novel serological biomarkers for the severity of disease and clinical outcomes

**Figure 2:**
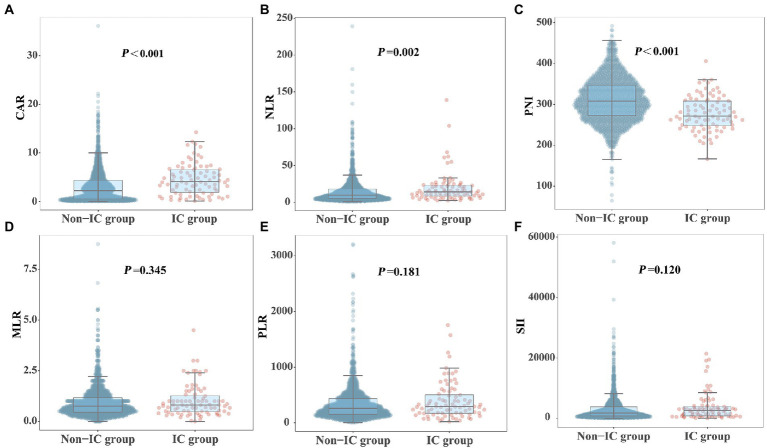
Comparison of novel serological biomarkers between IC group and non-IC group. **(A)** CAR, **(B)** NLR, **(C)** PNI, **(D)** MLR, **(E)** PLR, **(F)** SII. Data were presented as medians (IQR). Statistical significance was calculated by Mann–Whitney *U* test. *P* values indicated differences between patients in IC and non-IC group.

**Figure 3:**
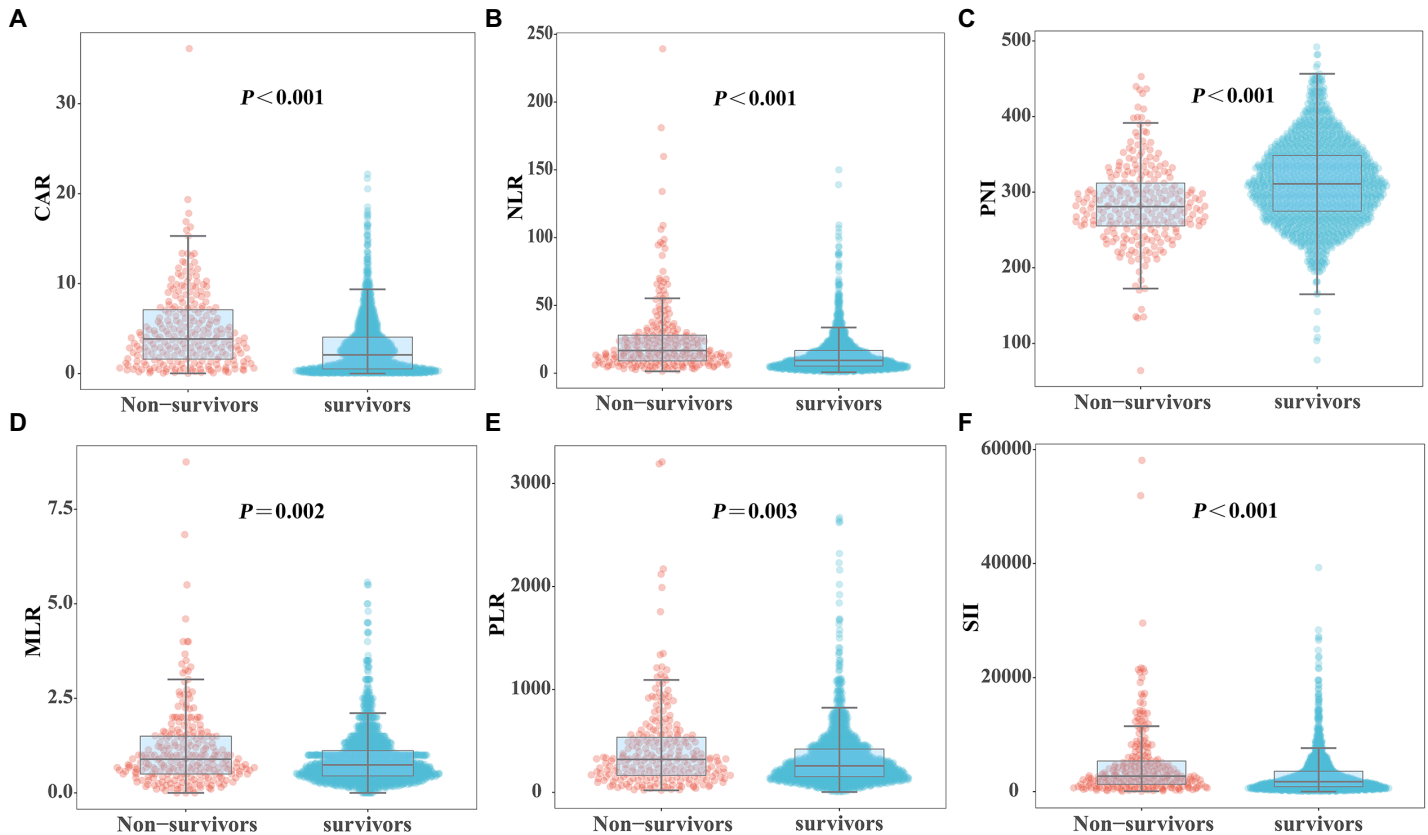
Comparison of novel serological biomarkers between survivors and non-survivors groups. **(A)** CAR, **(B)** NLR, **(C)** PNI, **(D)** MLR, **(E)** PLR, **(F)** SII. Data were presented as medians (IQR). Statistical significance was calculated by Mann–Whitney *U* test. *p* values indicated differences between patients in non-survivors and survivors group.

**Figure 4:**
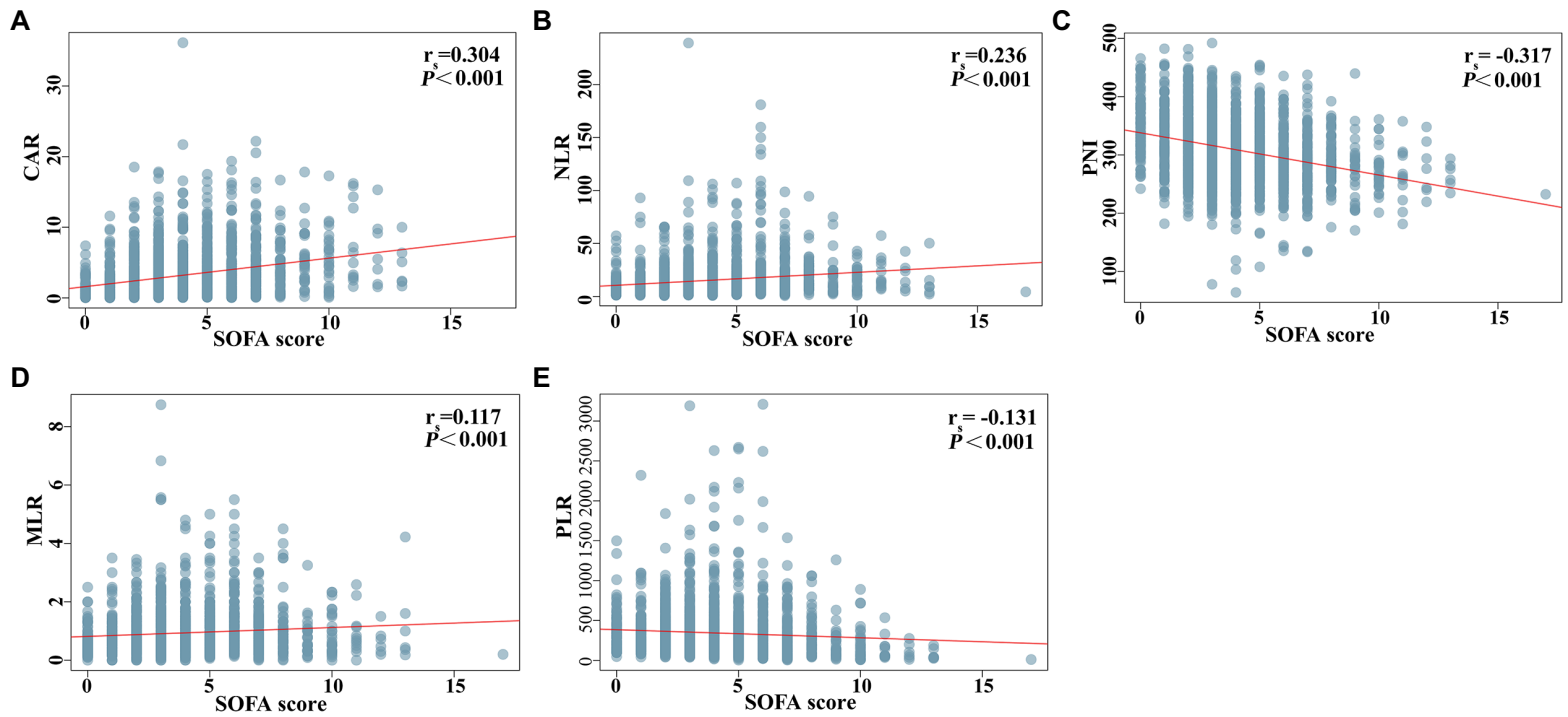
Correlation analysis between novel serological biomarkers and SOFA score. **(A)** CAR, **(B)** NLR, **(C)** PNI, **(D)** MLR, **(E)** PLR. Correlations between variables were analyzed with Spearman’s coefficients.

Figure 2 shows the differences in the novel serological biomarker levels between the IC and non-IC groups. CAR [4.08 (1.78–6.62) vs. 2.23 (0.55–4.36), *p* < 0.001] and NLR [14.50 (8.67–23.60) vs. 10.02 (5.58–18.50), *p* = 0.002] were significantly higher and PNI [272 (247–310) vs. 307 (272–346), *p* < 0.001] was much lower in the IC group than in the non-IC group. The MLR, PLR, and SII levels did not differ between the two groups. The total study population was divided into the survivor and non-survivor groups according to the 30-day mortality rates. Then we found CAR [3.78 (1.62–7.10) vs. 2.28 (0.55–3.96), *p* < 0.001], NLR [16.67 (9.00–28.00) vs. 9.40 (5.20–16.66), *p* < 0.001], MLR [0.89 (0.50–1.50) vs. 0.74 (0.45–1.13), *p* = 0.002], PLR [320.00 (162.50–538.00) vs. 257.50 (151.74–420.95), *p* = 0.003], and SII [2725.00 (1273.47–5382.55) vs. 1746.00 (868.65–3587.25), *p* < 0.001] were significantly higher in non-survivors than in survivors, while PNI [281.00 (254.97–312.50) vs. 311.00 (274.85–348.68), *p* < 0.001] was significantly lower in non-survivors than in survivors (Figure 3). Correlation analysis was performed to correlate the SOFA scores with novel biomarker levels. As shown in Figure 4, CAR, NLR, and MLR were positively correlated with SOFA scores (*p* < 0.001), and PNI and PLR were inversely correlated with SOFA scores (*p* < 0.001).

### Development of the scoring system

The results of univariate and multivariate logistic regression analyzes in the development cohort are presented in Tables 2, 3. Variables demonstrating possible risk factors for IC were transformed from continuous variables into categorical variables using cut-off values generated from ROC analysis with the best Youden index. The best cutoff points for NLR, PNI, CAR, and SOFA scores were 8.65, 281.25, 4.31, and 4.5, respectively, for predicting IC. Thus, high NLR, low PNI, high CAR, and high SOFA scores were defined as ≥8.65, < 281.25, ≥4.31, and ≥ 4.5, respectively. Sixteen significant factors in the univariate analysis, including DM, sepsis, mean arterial pressure, oxygenation index, invasive mechanical ventilation, vasopressor, CRRT, TPN, urinary catheter, CVC, broad-spectrum antibiotics, SOFA score, BDG positivity, high NLR, low PNI, and high CAR, were included in the multivariate forward stepwise logistic regression analysis. Multivariate logistic regression analysis showed that sepsis (OR: 2.065, 95% CI: 1.073–3.973, *p* = 0.030), TPN (OR: 2.034, 95% CI: 1.015–4.078, *p* = 0.045), broad-spectrum antibiotics (OR: 3.376, 95% CI: 1.231–9.264, *p* = 0.018), BDG-positivity (OR: 14.320, 95% CI: 7.653–26.795, *P* < 0.001), high NLR (OR: 2.608, 95% CI: 1.212–5.609, *p* = 0.014), high CAR (OR: 1.952, 95% CI: 1.045–3.646, *p* = 0.036), and high SOFA score (OR: 1.922, 95% CI: 1.016–3.634, *p* = 0.045) were risk factors for IC in the final model. Then, we divided each β coefficient by the smallest β coefficient value and assigned the rounded integer to each variable, constructing a scoring system (Table 3).

**Table 2:** Univariate analysis for IC-associated risk factors in the development cohort.

Variables	Univariate analysis	
	OR (95% CI)	*p* Value
Diabetes mellitus	1.724 (1.002–2.966)	0.049
Sepsis	5.494 (3.255–9.272)	<0.001
Mean arterial pressure (mmHg)	0.979 (0.963–0.995)	0.009
Oxygenation index (mmHg)	0.995 (0.993–0.998)	0.001
Invasive mechanical ventilation	4.935 (2.692–9.047)	<0.001
Vasopressor	4.239 (2.484–7.234)	<0.001
CRRT	3.675 (2.011–6.715)	<0.001
TPN	3.482 (1.954–6.206)	<0.001
Urinary catheter	4.549 (2.146–9.642)	<0.001
CVC	4.739 (2.761–8.134)	<0.001
Broad-spectrum antibiotics	5.311 (2.270–12.425)	<0.001
SOFA score	1.234 (1.130–1.349)	<0.001
BDG positivity	15.215 (8.723–26.539)	<0.001
NLR	1.016 (1.006–1.026)	0.002
PNI	0.988 (0.983–0.992)	<0.001
CAR	1.100 (1.045–1.158)	<0.001

OR, odds ratio; CI, confidence interval.

**Table 3:** Multiple logistic regression model and weighed point assignment.

Variables	β	*p* Value	Exp (β)	Exp (β) 95% CI	points
Sepsis	0.725	0.030	2.065	1.073–3.973	1
TPN	0.710	0.045	2.034	1.015–4.078	1
Broad spectrum antibiotics	1.217	0.018	3.376	1.231–9.264	2
BDG positivity	2.662	<0.001	14.320	7.653–26.795	4
High NLR (≥8.65)	0.958	0.014	2.608	1.212–5.609	1
High CAR (≥4.31)	0.669	0.036	1.952	1.045–3.646	1
High SOFA score (≥4.5)	0.653	0.045	1.922	1.016–3.634	1

NLR ≥ 8.65, CAR ≥ 4.31 and SOFA score ≥ 4.5 were identified using cut-off value generating from ROC analysis, respectively. The weighed point of each variable was defined by corresponding β coefficient which was divided each beta coefficient by the absolute value of the smallest beta coefficient, round to the nearest integer.

### Validation of the scoring system

**Figure 5:**
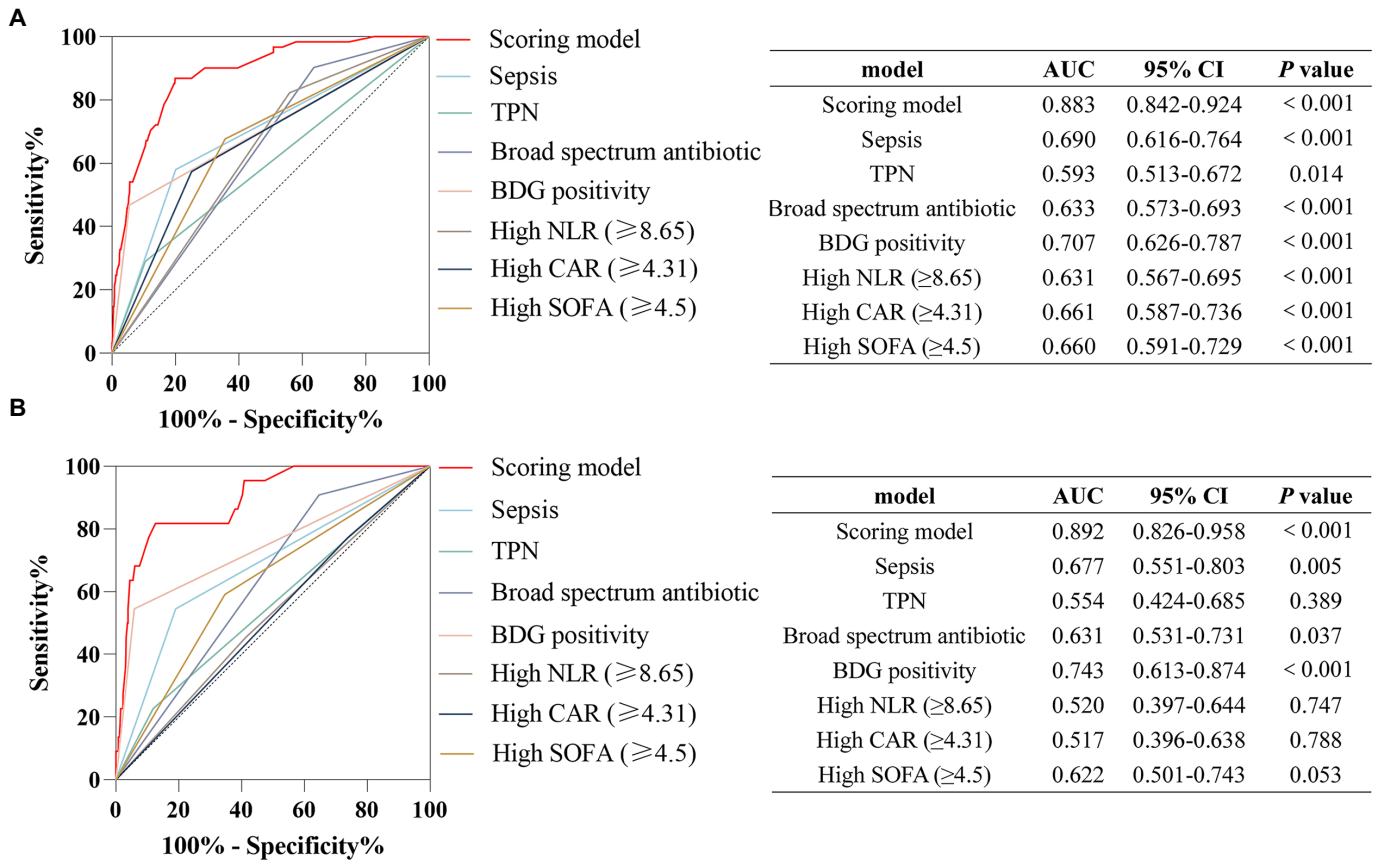
Receiver operating characteristic curves of the predictive model and single variables for the prediction of IC risk in **(A)** the development cohort, **(B)** the validation cohort.

**Figure 6:**
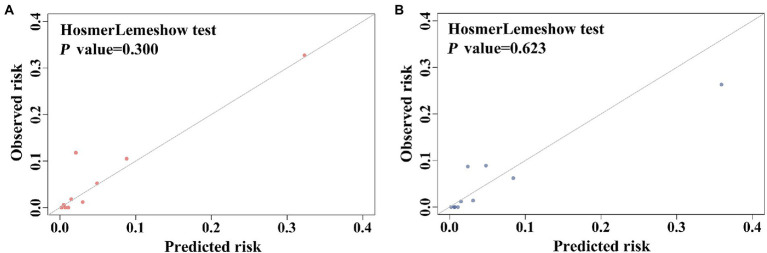
Calibration performance of the scoring model in **(A)** the development cohort, **(B)** the validation cohort.

The predictive scoring system performed well with an AUC of 0.883 (95% CI: 0.842–0.924, *p* < 0.001) in the development cohort and an AUC of 0.892 (95% CI, 0.826–0.958, *p* < 0.001) in the validation cohort (Figure 5). Moreover, the predictive power of the overall model was superior to that of a single variable in both the development and validation cohorts (Figure 5). The scoring system also showed good calibration in both the development and validation cohorts (Hosmer-Lemeshow *p* = 0.300 and 0.623, respectively), as depicted in the calibration plot (Figure 6). Furthermore, the estimated risk of IC was highly correlated with the observed risk (Spearman’s contingency coefficient = 0.723, *p* < 0.001).

### Clinical performance of the scoring system

**Figure 7:**
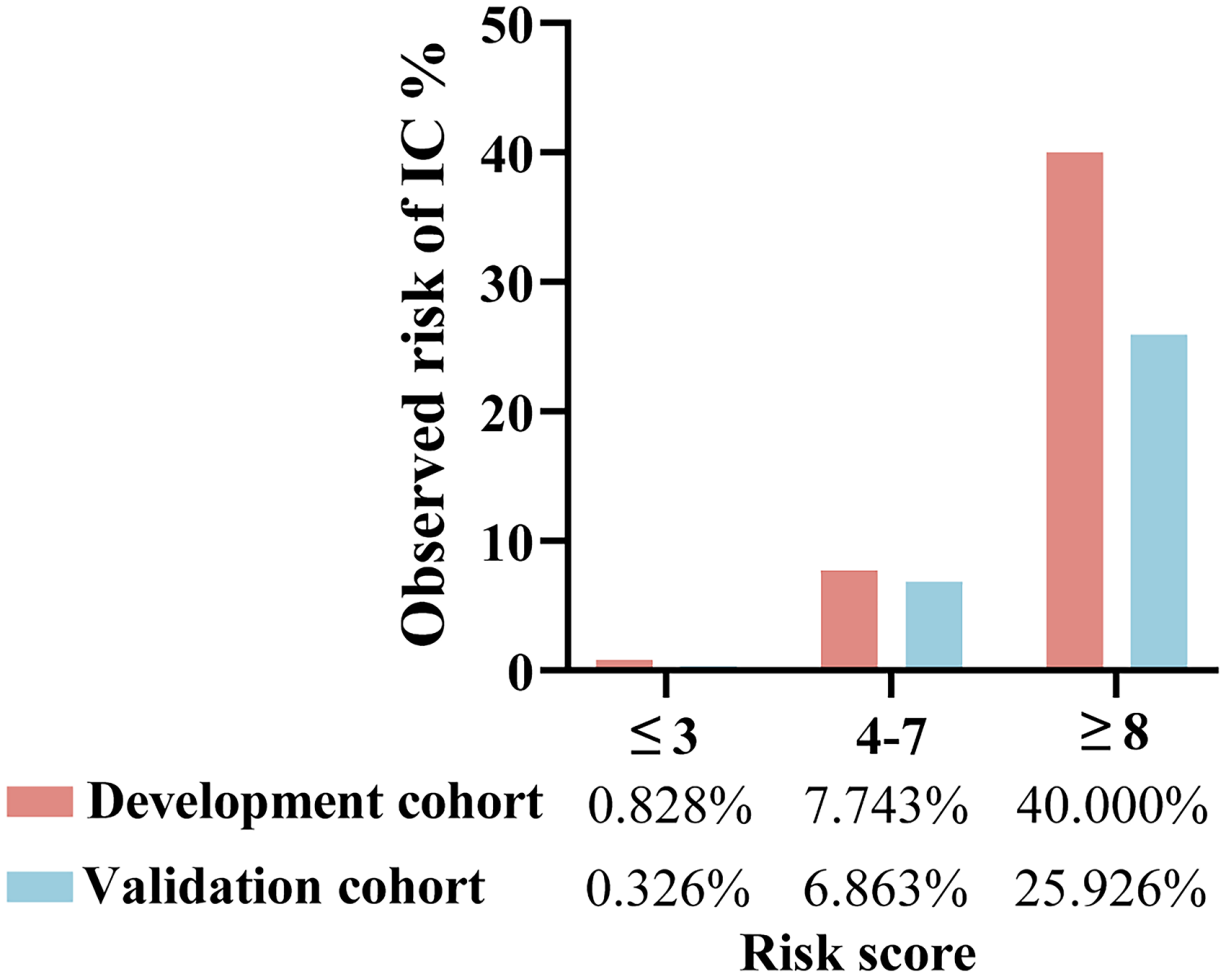
Risk groups according to risk score of the predictive scoring system and observed risk of IC in the development and validation cohorts. ≤3 points refers to low-risk group; 4–7 points refers to medium-risk group; ≥8 points refers to high-risk group.

The patients were divided into low-(≤3 points), medium-(4–7 points), and high-risk (≥8 points) groups. In the development cohort, 725 cases were included in the low-risk group, with six developing IC (0.828%). A total of 452 cases were included in the medium-risk group, with 35 developing IC (7.743%). Fifty cases were included in the high-risk group, with 20 developing IC (40.000%). In the validation cohort, the low-risk group comprised 307 patients, with one developing IC (0.326%). The medium-risk group comprised 204 patients, with 14 developing IC (6.863%). The high-risk group comprised 27 patients, with seven developing IC (25.926%; Figure 7). Table 4 presents the detailed clinical implications of the scoring system for different score thresholds. According to the ROC analysis, the cut-off point of the scoring system was 4.5 points. The sensitivity and specificity for ≥4 points were 91.57 and 60.94%, respectively; the PPV and NPV were 10.37 and 99.32%, respectively, with an LR^+^ of 2.34 and LR^−^ of 0.14. The sensitivity and specificity of ≥5 points were 83.13 and 78.40%, respectively; the PPV and NPV were 15.94 and 98.95%, respectively, with an LR^+^ of 3.84 and LR^−^ of 0.22.

**Table 4:** Performance value of the scoring model at different score thresholds.

Predictive score	*n* (%)	Sensitivity (%)	Specificity (%)	PPV (%)	NPV (%)	LR^+^	LR^−^
≥1	1,542 (83.76%)	100.00	13.26	5.38	100.00	1.15	0.00
≥2	1,343 (72.95%)	98.80	25.03	6.11	99.76	1.32	0.05
≥3	1,059 (57.52%)	98.80	41.91	7.74	99.86	1.70	0.03
≥4	733 (39.82%)	91.57	60.94	10.37	99.32	2.34	0.14
≥5	433 (23.52%)	83.13	78.40	15.94	98.95	3.84	0.22
≥6	244 (13.25%)	63.86	88.64	21.72	98.03	5.62	0.41
≥7	127 (6.90%)	46.99	94.77	30.71	97.31	8.98	0.56
≥8	77 (4.18%)	32.53	97.03	35.06	96.68	10.94	0.70
≥9	43 (2.23%)	21.69	98.51	41.86	96.23	14.59	0.79
≥10	19 (1.03%)	12.05	99.46	52.63	95.82	22.52	0.88

### Diagnostic value of the scoring system

**Figure 8:**
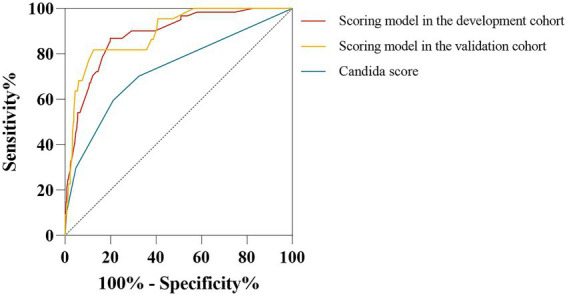
Comparison of receiver operating characteristic curves for IC risk between the scoring model and *Candida* score. The AUC of the scoring model was similar between the development cohort and validation cohort (0.883 vs. 0.892, *p* = 0.825). AUC of the scoring model was higher than the *Candida* score (0.883 vs. 0.730, *Z* statistic = 4.229, *p* < 0.001; 0.892 vs. 0.730, *Z*-statistic = 3.592, *p* < 0.001).

Previous studies have shown that a high *Candida* score is associated with an increased risk of IC and is commonly used for diagnosing IC (Leon et al., 2009; Barantsevich and Barantsevich, 2022). We generated ROC curves to evaluate the diagnostic value of the *Candida* score and the predictive scoring system for the early diagnosis of IC among immunocompetent critically ill patients. The AUCs were 0.730 (95% CI: 0.667–0.792, *p* < 0.001) for the *Candida* score and 0.883 (95% CI: 0.842–0.924, *p* < 0.001) for the scoring system (Figure 8). As shown in Figure 8, the AUC of the scoring model was larger than that of the *Candida* score (0.883 vs. 0.730, z-statistic, 4.229, *p* < 0.001).

## Discussion

At present, the guidelines and studies related to IC are mainly focused on people with immunodeficiency but less on immunocompetent populations (Donnelly et al., 2020). However majorities of critically ill patients are immunocompetent (Bassetti et al., 2021). Thus, early diagnosis of IC remains challenging among immunocompetent, critically ill patients, resulting in delayed antifungal treatment and increased mortality. Clinicians urgently need a tailored and prompt diagnostic method to recognize IC in ICU patients and instruct timely antifungal therapy. In this study, we found that new biomarkers, CAR, NLR, and PNI, were significantly altered in patients with IC. Furthermore, these biomarkers were significantly associated with disease severity and prognosis of ICU patients. Finally, we developed a scoring system for the early diagnosis of IC in immunocompetent patients in the ICU. The scoring system, based on sepsis, TPN, broad-spectrum antibiotics, high SOFA score, BDG positivity, high NLR, and high CAR, had a good identifiable value for IC, with an AUC of 0.883.

The burden of invasive fungal disease in ICU patients is increasing, of which 80% is caused by *Candida* species (Bassetti et al., 2017). In this study, the incidence of IC in immunocompetent ICU patients was 4.56% (84/1841), similar to previous studies (Zhang et al., 2022). The all-cause mortality of IC is reported to be approximately 49% (Kollef et al., 2012). However, the overall mortality rate of IC in our study population was 21.4% (18/84), which was lower than the published data. This may be related to the exclusion of immunodeficient patients in our study. In addition, we showed that patients with IC had a significantly higher disease severity score, 30-day mortality rate, hospitalization expenses, and longer hospital and ICU lengths of stay than those non-IC patients. Therefore, patients with IC have a higher mortality and socioeconomic burden.

Delayed antifungal treatments could further increase the mortality of ICU patients with IC (Martin-Loeches et al., 2019). Therefore, timely diagnosis of IC is crucial to guide targeted antifungal therapy and to improve the prognosis of ICU patients. Nevertheless, early diagnosis of IC in the ICU setting remains difficult. First, tissue biopsy, the gold standard for diagnosing IC, is difficult to accomplish in ICU patients because of their unstable vital signs and/or coagulation disorders. Second, noninvasive diagnostic methods have their respective limitations. For example, BDG is a cell wall component of *Candida*, which can be continuously released into blood in the process of IC (Clancy and Nguyen, 2018; Yoshida, 2021). So the detection of BDG is a common diagnostic method for IC. However, the detection methodologies are diverse, such as turbidimetry, colorimetry, or chemiluminescence immunoassay (Clancy and Nguyen, 2018; Yoshida, 2021). And colorimetry includes endpoint colorimetry and dynamic colorimetry, while turbidimetry also includes endpoint turbidimetry and dynamic turbidimetry. So the thresholds of BDG testing are different, ranging from 11 pg./ml to 95 pg./ml, making it difficult to reach a consensus (White et al., 2019; Bamba et al., 2021; Lamoth et al., 2021; Lass-Florl et al., 2021). Additionally, BDG detection has a high false-positive rate (Karageorgopoulos et al., 2011; Haydour et al., 2019) and can be influenced by blood product perfusion, *Candida* spp. colonization, and disturbed GI mucosa (Logan et al., 2020).

Critically ill IC patients exhibit extremely severe systemic inflammation and poor nutritional status. Novel markers derived from a combination of two or more laboratory indicators, such as NLR, PLR, MLR, CAR, and SII, represent inflammation and immune responses (Xue et al., 2020). Meanwhile, PNI symbolizes the nutritional status (Xue et al., 2020). Higher levels of NLR, PLR, MLR, CAR, and SII and lower levels of PNI are correlated with the severity of various diseases, such as COVID-19, cancer, and rheumatic diseases (Gasparyan et al., 2019; Fois et al., 2020; Huang et al., 2020; Nost et al., 2021). In our study, we found that NLR and CAR were higher in patients with IC, whereas PNI was lower. Correlation analysis showed that CAR, NLR, and MLR were positively correlated with SOFA scores, while PNI and PLR were reversed. Furthermore, CAR, NLR, MLR, PLR, and SII were higher and PNI was lower in the non-survivor group. Therefore, our study revealed that these new biomarkers are closely related to IC, and the disease severity and clinical outcomes of ICU patients. In addition, multivariate logistic regression analysis showed that high NLR (OR: 2.608, 95% CI: 1.212–5.609, *p* = 0.014) and high CAR (OR: 1.952, 95% CI: 1.045–3.646, *p* = 0.036) were independent risk factors for IC. Therefore, NLR and CAR may be potential biomarkers for IC.

The most common mycosis in ICU patients is IC (Pappas et al., 2018). The risk factors for IC are closely related to the patient’s physical condition, disease, and iatrogenic treatment. At present, common risk factors for IC include long-term use of corticosteroids, broad-spectrum antibiotics, TPN, indwelling CVC, any type of dialysis, invasive mechanical ventilation, and surgery (Pappas et al., 2018; Martin-Loeches et al., 2019). ICU patients are more likely to be exposed to these risk factors, which leads to a higher incidence of IC in ICU patients. Our study also demonstrated that TPN and broad-spectrum antibiotic were independent risk factors for IC. As for TPN, lipid and glucose contained in nutrient solutions could propagate biofilm formation of *Candida* spp. (Kuwahara et al., 2010). Furthermore, blocked intestinal peristalsis leads to changes in the intestinal epithelial barrier function and promotes the translocation of intestinally attached *Candida* into the bloodstream (Pierre, 2017). Broad-spectrum antibiotics would increase the colonization rate of *Candida* spp. and change the interaction between bacteria and fungi, leading to enhanced pathogenicity of *Candida* (Guinan et al., 2019). In addtion, our study found that sepsis is a risk factor for IC. Previous studies have suggested that patients with sepsis are more likely to receive long-term antibiotics and invasive medical devices, which are definite risk factors for IC (Meng et al., 2017; Wang et al., 2021). Moreover, sepsis induces immune paralysis, predisposing the host to *Candida* infection (Jung et al., 2021).

Based on traditional BDG, combined biomarker NLR, CAR, and classical clinical risk factors (sepsis, TPN, broad-spectrum antibiotic, and SOFA), we established a bedside risk-scoring model for predicting the risk of IC. The model performed excellently with an AUC of 0.883 in the development cohort and was well validated in the validation cohort (AUC = 0.892). The estimated score was highly positively related to the actual risk of IC. Furthermore, the discriminative value of our scoring model was higher than that of the *Candida* scoring system. The above parameters included in the model are available, economical, and reliable in clinical practice and are usually included in routine admission examinations among ICU patients. Thus, this scoring model can be extensively used, even in grassroots hospitals. Therefore, it is convenient for physicians to evaluate the risk of IC for patients at the bedside.

However, our study has some limitations. First, this was a single-center retrospective study; therefore, we lacked another hospital validation cohort to validate our scoring system. However, the validation in our study exhibited a satisfactory AUC and calibration in both the development and validation cohorts, even with a higher discriminative value than the *Candida* score. Second, although the scoring system can early and accurately diagnose IC among ICU patients, the clinical benefits of the clinical management strategy based on our scoring system need to be confirmed. In the future, a larger prospective cohort involving multicenter hospitals is needed to evaluate the clinical performance of our scoring system.

## Conclusion

In conclusion, our results demonstrated that NLR, CAR, and PNI were significantly associated with IC and disease severity in immunocompetent critically ill patients. We established a parsimonious score based on NLR, CAR, clinical risk factors (sepsis, TPN, broad-spectrum antibiotic), SOFA, and BDG, which can diagnose IC in immunocompetent ICU patients early and accurately to guide clinical physicians to initiate antifungal therapy at an early stage and improve the prognosis of IC.

## Data availability statement

The raw data supporting the conclusions of this article will be made available by the authors, without undue reservation.

## Ethics statement

The studies involving human participants were reviewed and approved by the Ethics Committee of the Xiangya Hospital, Central South University (No. 202104005). Written informed consent for participation was not required for this study in accordance with the national legislation and the institutional requirements.

## Author contributions

YL and PP conceived, supervised the study and approved the final version of manuscript. YL revised the manuscript. PP provided funds. WL and GC analyzed data, prepared figures, tables and drafted the manuscript. FL and HY provided codes for statistical analysis. YC, RL, CS, and HL collected data. All authors read and approved the final manuscript.

## Funding

This study was supported by Key R&D Program of Hunan Province (No. 2022SK2038), Project Program of National Clinical Research Center for Geriatric Disorders (Xiangya Hospital, Grant No. 2020LNJJ05), National Natural Science Foundation of China (No. 82000089, No. 82100100 and No. 82200099), Hunan Natural Science Youth Foundation (No.2022JJ40810 and No. 2022JJ40775), The National Key Clinical Specialist Construction Programs of China (No. z047-02), Scientific research project of Hunan Health Commission (No. 202103020612) and Science and Technology Projects in Guangzhou (No. 202201010012).

## Conflict of interest

The authors declare that the research was conducted in the absence of any commercial or financial relationships that could be construed as a potential conflict of interest.

## Publisher’s note

All claims expressed in this article are solely those of the authors and do not necessarily represent those of their affiliated organizations, or those of the publisher, the editors and the reviewers. Any product that may be evaluated in this article, or claim that may be made by its manufacturer, is not guaranteed or endorsed by the publisher.
