# Supplementary material for: A scoring system based on novel biomarkers and clinical risk factors to predict invasive candidiasis in immunocompetent critically ill patients
Source: Front Microbiol. 2023 Mar 9;14:1097574. doi: 10.3389/fmicb.2023.1097574 (PMC10033536; doi:10.3389/fmicb.2023.1097574)
Supplement: Supplementary file 1 [file Table_1.DOCX]

| **Table S1. Comparison of basic characteristics between the development and validation cohort** | | | |
| --- | --- | --- | --- |
| **Variables** | **Development Cohort (n=1289)** | **Validation cohort**  **(n=552)** | ***P* value** |
| **Age,** **median (IQR), years** | 67 (55.50-74.00) | 67 (56.00-74.00) | 0.872 |
| **Male, n (%)** | 938 (73.77%) | 404 (73.19%) | 0.853 |
| **Smoke history, n (%)** | 766 (59.43%) | 334 (60.51%) | 0.665 |
| **Comorbidities, n (%)** |  |  |  |
| COPD | 392 (30.41%) | 172 (31.16%) | 0.750 |
| Diabetes mellitus | 302 (23.43%) | 125 (22.64%) | 0.715 |
| Sepsis | 283 (21.96%) | 113 (20.47%) | 0.478 |
| Pulmonary tuberculosis | 198 (15.36%) | 80 (14.49%) | 0.634 |
| Bronchiectasis | 160 (12.41%) | 73 (13.22%) | 0.631 |
| Chronic renal failure | 154 (11.95%) | 70 (12.68%) | 0.659 |
| Interstitial lung disease | 133 (10.32%) | 48 (8.70%) | 0.284 |
| Rheumatic immune disease | 125 (9.70%) | 51 (9.24%) | 0.759 |
| Lung cancer | 124 (9.62%) | 48 (8.70%) | 0.533 |
| Other solid tumor | 93 (7.21%) | 38 (6.88%) | 0.800 |
| Asthma | 58 (4.50%) | 23 (4.17%) | 0.750 |
| Hepatic failure or cirrhosis | 26 (2.02%) | 13 (2.36%) | 0.645 |
| **Vital signs on admission to ICU** |  |  |  |
| Body temperature (℃) | 36.80 (36.50-37.00) | 36.80 (36.50-37.00) | 0.872 |
| Respiratory rate (times per minute) | 23 (21-26) | 23 (20-26) | 0.478 |
| Heart rate (times per minute) | 92.00 (80.00-105.00) | 91.50 (80.00-104.00) | 0.641 |
| Mean arterial pressure (mmHg) | 91.67 (81.33-101.67) | 89.50 (81.00-100.00) | 0.188 |
| Oxygenation index (mmHg) | 231.79 (144.00-308.30) | 221.18 (148.61-320.00) | 0.384 |
| **Life-sustaining treatments, n(%)** |  |  |  |
| Non-invasive mechanical ventilation | 254 (19.71%) | 104 (18.84%) | 0.668 |
| Invasive mechanical ventilation | 551 (42.75%) | 253 (45.83%) | 0.221 |
| Vasopressor | 408 (31.65%) | 181 (32.79%) | 0.639 |
| CRRT | 122 (9.46%) | 48 (8.70%) | 0.598 |
| TPN | 147 (11.40%) | 68 (12.32%) | 0.576 |
| Surgery | 80 (6.21%) | 44 (7.97%) | 0.166 |
| **Indwelling catheter, n (%)** |  |  |  |
| Urinary catheter | 787 (61.06%) | 342 (61.96%) | 0.683 |
| CVC | 399 (30.95%) | 184 (33.33%) | 0.303 |
| **Drug therapy, n (%)** |  |  |  |
| High-dose of corticosteroid | 548 (42.51%) | 228 (41.30%) | 0.630 |
| Broad-spectrum antibiotics | 838 (65.01%) | 363 (65.76%) | 0.757 |
| **Disease severity score** |  |  |  |
| SOFA score, median (IQR) | 4 (2-5) | 4 (2-5) | 0.943 |
| **Fungal infection marker** |  |  |  |
| BDG positivity, n (%) | 96 (7.45%) | 43 (7.79%) | 0.799 |
| **Novel serological markers** |  |  |  |
| NLR | 10.00 (5.60-18.41) | 10.24 (5.44-18.75) | 0.611 |
| PLR | 259.09 (150.00-433.63) | 270.76 (160.00-440.00) | 0.157 |
| MLR | 0.75 (0.46-1.20) | 0.75 (0.46-1.14) | 0.778 |
| PNI | 304.25 (270.13-344.55) | 311.25 (270.63-348.13) | 0.371 |
| SII | 1850.72 (870.44-3725.25) | 1927.62 (901.25-4119.03) | 0.265 |
| CAR | 2.44 (0.63-4.38) | 2.45 (0.60-4.34) | 0.689 |
| **Clinical outcomes** |  |  |  |
| IC, n (%) | 62 (4.81%) | 22 (3.99%) | 0.437 |
| 30-day mortality, n (%) | 163 (12.65%) | 86 (15.58%) | 0.092 |
| Hospital duration of stay (days) | 9 (6-16) | 9 (6-16) | 0.923 |
| ICU length of stay (days) | 7.00 (4.00-10.75) | 7.00 (4.00-10.00) | 0.602 |
| Hospitalization expenses | 55725.21 (33846.87-107488.50) | 57573.48 (35188.58-106385.05) | 0.781 |
| Data were presented as median (IQR) and n (%). *P* values were calculated by Mann-Whitney U test, or Chi-square test, as appropriate. *P* values indicated differences between the IC and non-IC patients. Abbreviations: COPD, chronic obstructive pulmonary disease; CRRT, continuous renal replacement therapies; TPN, total parenteral nutrition; CVC, central venous catheter; IVIG, intravenous immunoglobulin; SOFA, Sequential Organ Failure Assessment; BDG, 1,3-β-D-glucan; NLR, neutrophil-to-lymphocyte ratio; PLR, platelet-to-lymphocyte ratio; MLR, monocyte-to-lymphocyte ratio; PNI, prognostic nutritional index; SII, systemic immune-inflammatory index; CAR, C-reactive protein-to-albumin ratio; IC, invasive candidiasis; ICU, intensive care unit; IQR, interquartile range. | | | |
